# Results of Pyrocarbon Disc Interposition Compared to Trapeziectomy with Ligament Reconstruction and Tendon Interposition

**DOI:** 10.1097/PRS.0000000000011038

**Published:** 2024-07-24

**Authors:** Cecile M. C. A. van Laarhoven, Simone J. A. Donners, Constance J. H. C. M. van Laarhoven, Joris Teunissen, Luc Bieckmann, Arnold H. Schuurman, Brigitte E. P. A. van der Heijden

**Affiliations:** Utrecht, Rotterdam, ‘s-Hertogenbosch, and Nijmegen, the Netherlands; From the 1 University Medical Center Utrecht, Utrecht University; 2 Erasmus Medical Center, Erasmus University; 3 Jeroen Bosch Hospital; 4 Radboud University Medical Center, Radboud University.

## Abstract

**Background:**

To compare pyrocarbon disc interposition arthroplasty (PDI) with trapeziectomy plus ligament reconstruction tendon interposition (LRTI), the authors assessed whether PDI resulted in a higher pinch strength, and compared grip strength, range of motion (ROM), patient-reported outcomes, satisfaction, and complications between the approaches.

**Methods:**

Because of scarcity of preoperative hand measurements, the authors performed a descriptional cross-sectional cohort study of patients operated on between 2006 and 2014, with a minimum of 5 years of follow-up. Patients were treated with PDI or LRTI. The authors determined key pinch strength as the primary outcome, followed by tip and tripod pinch, grip strength, palmar abduction and opposition, Michigan Hand Outcomes Questionnaire (MHQ) and Patient-Reported Hand and Wrist Evaluation (PRWHE) scores, satisfaction level, and complications. Propensity score matching was used to match the study groups on demographic variables. A ratio of 2:1 was used, resulting in inclusion of 62 (of 154) PDI and 31 (of 31) LRTI thumbs.

**Results:**

Patients in the PDI group showed stronger key and tip pinch strength than did patients in the LRTI group (*P* = 0.027 and *P* = 0.036, respectively). Tripod pinch, grip strength, and ROM were equal between the groups. MHQ and PRWHE were comparable, with higher satisfaction levels in the PDI group. Eight patients with PDI were converted to LRTI because of pain.

**Conclusions:**

This study confirmed the hypothesis that key and tip pinch strength is stronger after PDI compared with LRTI for first carpometacarpal joint osteoarthritis. Both techniques have comparable outcomes considering patient-reported outcome (MHQ and PRWHE), ROM, and complications.

**CLINICAL QUESTION/LEVEL OF EVIDENCE:**

Therapeutic, III.

Osteoarthritis of the first carpometacarpal (CMC-1) joint is a degenerative disease most commonly affecting postmenopausal woman.^1,2^ Surgical intervention is indicated in case of advanced progression of disease involving symptomatic patients with impairment in range of motion (ROM) and strength or pain.^3,4^ Despite extensive research comparing numerous operative strategies over the past decades, no method has been proven to be superior concerning reduction of pain or improvement in physical function or patient-reported outcome measures (PROMs).^5^

Trapeziectomy usually is performed, with or without a tendonplasty.^6–10^ An additional ligament reconstruction and tendon interposition plasty (LRTI) is performed to enhance stability and prevent proximal migration and subsequent grinding of the first metacarpal on the proximal hemitrapezium.^11^ However, it has been shown that an autologous tendon graft hardly maintains the height of the trapezial space, and postoperative outcomes are variable regarding strength and ROM.^12,13^

In patients with radiologic Eaton-Glickel stage II or III,^10^ a distal hemitrapeziectomy instead of a total trapeziectomy can be performed, saving the scaphotrapeziotrapezoidal (STT) joint. It is postulated that with the interposition of the pyrocarbon disc (PyroDisk; Integra LifeSciences Corporation), the shortcomings of autologous tendon graft can be overcome.^14^ This implant is a biarticular convex disc made of pyrocarbon, designed to resurface the damaged CMC-1 joint after hemitrapeziectomy. Interposition of the pyrocarbon disc can preserve thumb height because of minimal resection of the articular surface and, in contrast to a tendon graft, has mechanical properties similar to that of the cortical bone.^15^ Previous studies have shown good results in terms of pain relief, ROM, pinch, and grip strength after hemitrapeziectomy combined with pyrocarbon disc interposition (PDI).^14,16–19^ Polyethylene wear and metallosis have not been observed, presuming high survival rates of the implant.^20,21^

A recent retrospective short-term study comparing trapeziectomy with LRTI with PDI showed stronger key pinch in favor of PDI at 3-year follow-up.^22^ Because studies that compare long-term follow-up are lacking, we compared the long-term results of PDI arthroplasty with those of trapeziectomy combined with LRTI. Our hypothesis is that after long-term follow-up, PDI treatment for CMC-1 joint osteoarthritis will result in greater pinch strength compared with LRTI. In addition to pinch strength, we compared grip strength, ROM, PROMs, satisfaction, and complications.

## PATIENTS AND METHODS

### Study Design and Patients

**Fig. 1.**
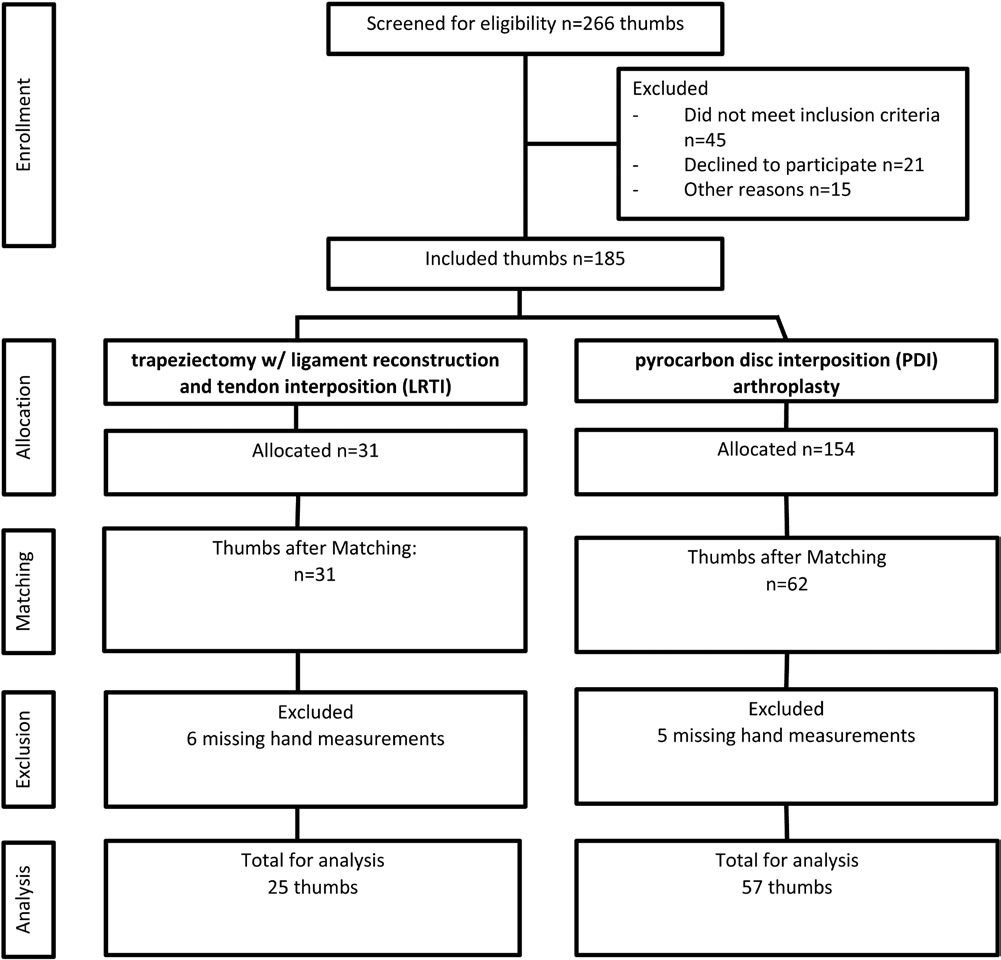
Overview of included study patients.

This study is part of our multicenter study project analyzing postoperative outcomes after CMC-1 joint osteoarthritis.^14^ Between 2006 and 2014, 4 surgeons in 2 centers operated on a total of 185 thumbs with either LRTI or PDI for CMC-1 joint osteoarthritis of Eaton-Glickel grade II or III (Fig. 1). After institutional review board approval was received, data for patients operated on with a PDI (PDI group) were gathered from our previous study.^14^ Data for patients undergoing LRTI (LRTI group) were obtained between the end of 2018 and the beginning of 2020. We designed a descriptive cross-sectional study with data obtained after a minimum 5-year follow-up. After written informed consent was obtained, PROM questionnaires were sent by email and patients were invited for clinical measurements and radiographs. Demographic characteristics, perioperative details, and complications were obtained by medical chart review. After consulting the patient on different treatment modalities, a shared decision-making process was carried out. Patients were treated with PDI or LRTI depending on patient and surgeon preference.

Inclusion criteria for the study were Eaton-Glickel stage II or III, nonresponsiveness to hand and plaster therapy for at least 3 months, and being operated on with LRTI or PDI with a minimum of 5 years after surgery. Exclusion criteria were involvement of the STT joint, CMC-1 joint surgery for hyperlaxity syndromes or systemic inflammatory arthritis, or less than 5-year follow-up. When necessary, computed tomography imaging was used to exclude STT involvement in case a PDI was planned. STT involvement was also checked during surgery, independent of surgical technique used.

### Surgical Techniques

#### PDI Arthroplasty

**Fig. 2.**
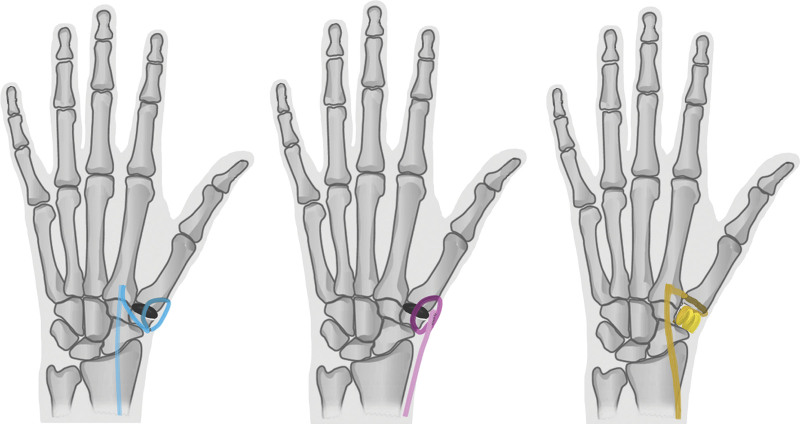
Illustration of techniques. (*Left*) PDI with FCR. (*Center*) PDI with APL. (*Right*) LRTI with FCR.

PDI was performed as described previously, with either the flexor carpi radialis (FCR)– or abductor pollicis longus (APL)–tendon strip to tighten the disc in its position in the trapezial space after a hemitrapeziectomy (Fig. 2, *left* and *center*).^23^ After harvesting one third to half of the FCR or APL, leaving the insertion intact, the tendon strip is consecutively passed through the bony tunnel created in trapezium, the disc and the bony tunnel created in the proximal metacarpal bone. The residual tendon is subsequently folded back and sutured to itself and the periosteum of the metacarpal base. Thereafter, the tendon strip is incorporated with the capsular closure using absorbable sutures for additional fixation of the disc and augmentation of the capsule. The position of the disc is assessed with radiography during the procedure and a thumb spica cast immobilization is applied for 4 weeks. Hand therapy is focused on unloaded ROM during the first 4-week postimmobilization period, followed by loaded ROM exercises up to full loaded motion after 12 weeks postoperatively. After 12 weeks, movement and loading had no restrictions.

#### Trapeziectomy Combined with LRTI

The trapeziectomy is performed through the same dorsal approach as that of the PDI group. Between the interval of the extensor pollicis longus and extensor pollicis brevis, the capsule is exposed and incised. The CMC-1 joint is exposed and the trapezium is taken out piecemeal. The STT joint is inspected to confirm that is free from osteoarthritis. Thereafter, an osseous tunnel is created in the base of the first metacarpal from middorsal to central in the joint space. Half to one third of the FCR tendon is harvested, leaving the insertion intact, with 2 stab incisions on the volar side of the wrist. The tendon is then brought in the trapezial space and routed through the osseous tunnel from proximal central to distal dorsal, whereafter the tendon is wrapped around itself in the depth of the joint space and sutured to itself. This provides a suspension of the first onto the second metacarpal. The remaining FCR tendon strip is rolled up and placed in the void after trapeziectomy (Fig. 2, *right*). The joint capsule is closed for extra stability. Total removal of the trapezium and position of the suspension is checked with radiography. Immobilization and hand therapy protocol were similar for both study groups.

### Measurements

**Fig. 3.**
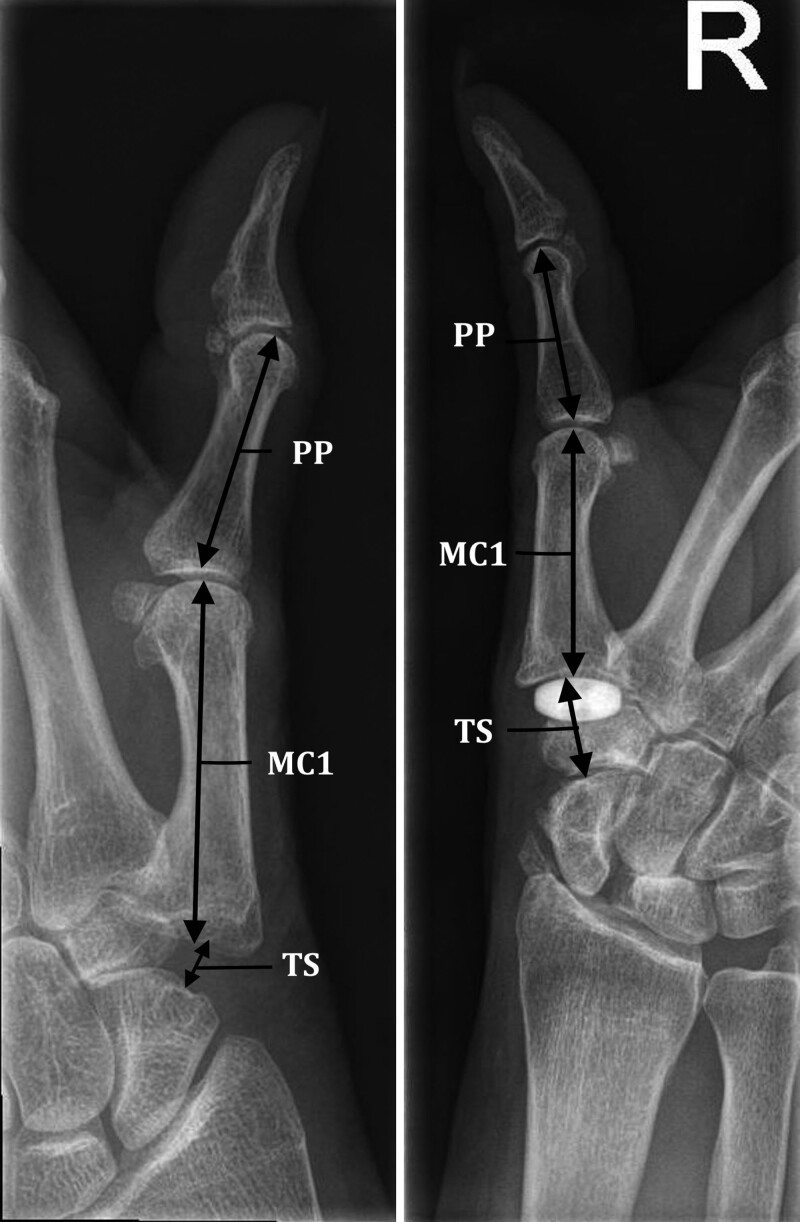
Example of measurement of thumb length on postoperative X-ray. (*Left*) After LRTI. (*Right*) After PDI.

The primary outcome of this study was key pinch strength, which was in line with the previous study by Oh et al.^22^ Secondary outcomes included tip pinch strength and tripod pinch strength. All 3 pinch strength variants were measured with a baseline pinch gauge (E-link H500 Hand Kit; Biometrics Ltd.). Furthermore, we collected data on grip strength (using a Jamar hydraulic hand dynamometer in position 2 [E-link H500 Hand Kit; Biometrics Ltd.]), thumb opposition (Kapandji score), and palmar abduction (using a Pollexograph^24,25^). For all these measures, the average of 3 consecutive measurements was used for analysis. In addition, patients completed the Michigan Hand Outcomes Questionnaire,^26^ Patient-Rated Wrist/Hand Evaluation,^27^ and a satisfaction questionnaire on a 10-point Likert scale (1, not satisfied at all; 10, excellent satisfaction). Radiographs (lateral, posteroanterior, and Bett view) of the treated hand were obtained at the last follow-up visit and compared with the immediate postoperative radiograph. We calculated total thumb height (Fig. 3) as a sum of the trapezial space and first metacarpal height and calculated a ratio with the proximal phalanx as the comparative standard.^28^

### Statistical Analysis

Because of the retrospective study design and the potential risk of confounding by indication bias, we used propensity score matching (PSM) to make the 2 groups (PDI and LRTI) more similar at baseline. The propensity score reflected the probability of receiving LRTI and was calculated using logistic regression modeling based on the following variables: age, sex, duration of follow-up (in years), whether the dominant side was treated or not, and whether the patient had surgery on the ipsilateral thumb. The propensity scores were then used to match patients from either group in a 1:2 ratio using the nearest-neighbor algorithm without replacement to increase precision. No caliper was prespecified. To assess for balance before and after PSM, standardized mean differences were calculated for each variable in the model. Standardized mean differences between 0 and 0.1 indicate good balance.^29^ PSM was performed in R Statistical Programming version 3.4.1 using the MatchIt package.

Kolmogorov-Smirnov tests and histograms were used to assess the distribution of continuous data. We reported descriptive statistics as means and standard deviations for normally distributed data, median and interquartile range for nonnormally distributed data, or absolute values and proportions (%) for categorical data. The 2 groups were analyzed using independent *t* test (normally distributed data), Mann-Whitney *U* test (nonnormally distributed data), or 2-proportion Z tests (categorical data). Patients in whom the pyrocarbon disc was removed before the long-term follow-up measurement were still included in all analyses to account for potential implant failure. *P* ≤ 0.05 was considered statistically significant. Statistical analyses were performed in R and SPSS 27.

### Power Analysis

To determine whether our study was sufficiently powered for all analyses, we performed post hoc sensitivity analyses. In these calculations, we used the number of available patients included in the respective analysis to calculate the effect size (Cohen *d*) that we could detect using a two-tailed distribution, a conventional power of 80%, and a significance level of 0.05. The magnitude of the effect size was interpreted as small (<0.20), moderate (0.20 to 0.80), or large (<0.80).^30^ The results of the sensitivity analyses are shown in Table 1.

**Table 1.** Detectable Effect Size of Each Outcome Measurement Comparison^a^

Outcome Measure	PDI Group (*n*)	LRTI Group (*n*)	Detectable Effect Size (Cohen *d*)	Interpretation
Key pinch, tip pinch, tripod pinch, Jamar, palmar abduction	57	25	0.68	Moderate
Kapandji opposition	56	25	0.68	Moderate
PRWHE total, function	58	28	0.65	Moderate
PRWHE pain	60	28	0.65	Moderate
MHQ total, aesthetics, pain, and work; average and treated hand ADL	60	29	0.64	Moderate
MHQ satisfaction	59	28	0.65	Moderate
Satisfaction with result	57	31	0.63	Moderate
Thumb length immediate follow-up	60	15	0.81	Large
Ratio immediate follow-up	60	16	0.80	Moderate
Thumb and ratio long-term follow-up	48	20	0.76	Moderate

ADL, activities of daily living; MHQ, Michigan Hand Outcomes Questionnaire; PRWHE, Patient-Rated Wrist/Hand Evaluation.

a To determine whether this study was sufficiently powered for the analyses and post hoc comparisons, we performed a post hoc sensitivity analysis. For these calculations, we used the number of included thumbs for the respective analysis to calculate the effect size (Cohen *d*) that we could detect using a two-tailed distribution, a conventional significance level of 0.05, and a power of 80%. The magnitude of the effect size was interpreted as small (<0.20), moderate (0.20 to 0.80), or large (<0.80).^30^

## RESULTS

A total of 154 thumbs treated with PDI and 31 thumbs treated with LRTI were considered for eligibility (Fig. 1). After PSM, 62 PDI thumbs and 31 LRTI thumbs were available for further analyses. Demographic characteristics before and after matching are presented in Table 2. PSM improved the balance in demographic characteristics between the 2 groups, although a small imbalance (standardized mean difference 0.2) remained, in which the average age in the PDI group was 2 years older. No further action was undertaken, as we did not consider this difference clinically relevant. The mean follow-up of both groups was 8 years (SD 2).

**Table 2.** Baseline Demographic Characteristics

Characteristics^a^	Before Matching				After Matching			
	PDI Group	LRTI Group	*P*	SMD	PDI Group	LRTI Group	*P*	SMD
No. of patients	154	31			62	31		
Age, yrs, mean (SD)	59 (9)	59 (7)	0.667	0.093	61 (9)	59 (7)	0.323	0.231
Male, *n* (%)	42 (27)	1 (3)	0.008	0.710	2 (3)	1 (3)	1.000	<0.001
Duration of follow-up, yrs, mean (SD)^b^	8 (2)	8 (2)	0.231	0.204	8 (2)	8 (2)	0.477	0.149
Dominant side treated, *n* (%)	63 (41)	12 (39)	0.978	0.045	25 (40)	12 (39)	1.000	0.033
Concomitant ipsilateral thumb surgery, *n* (%)	23 (15)	4 (13)	0.989	0.059	6 (10)	4 (13)	0.906	0.102

SMD, standardized mean difference.

a Characteristics before and after matching. Patients who underwent revision surgery (disc removal) are included in this analysis.

b Patients were invited for an evaluation with a minimum of 5 years of follow-up. The range of follow-up was 5 to 12 years.

Hand measurements are depicted in Table 3. Key and tip pinch strength was stronger in the PDI group (*P* = 0.027, *P* = 0.036, respectively); tripod pinch (*P* = 0.124), grip strength (*P* = 0.778), and ROM (palmar abduction *P* = 0.766, Kapandji *P* = 0.946) were comparable between groups. The PDI group showed better patient-rated aesthetics and activities of daily living for the treated hand, measured on Michigan Hand Questionnaire subscales, compared with the LRTI group (Table 4; *P* = 0.009). Thumb height and the thumb height ratio were both better preserved for the PDI group compared with the LRTI group, as is summarized in Table 5. At immediate follow-up (*P* = 0.001) as well as long-term follow-up (*P* < 0.001), the PDI group showed better thumb height (Table 5). Table 6 shows the complication rate in both groups. In 8 PDI-treated patients (12.9%), the pyrocarbon disc was removed and converted to a trapeziectomy with LRTI, because of persisting pain and STT arthritis (5 patients) and persisting pain without a specific cause (3 patients). No conversion surgery was performed after LRTI. No significant differences in complication rate were observed (*P* = 0.916).

**Table 3.** Hand Measurements^a^

Measurements, Treated Hand^b^	PDI after Matching	LRTI after Matching	*P*	% of Measurements Missing
No.	57^c^	25^d^		
Key pinch	4.20 (2.50–5.00)	2.90 (1.80–3.90)	0.027^e^	0.0
Tip pinch	3.00 (2.30–3.70)	2.20 (1.50–3.20)	0.036^e^	0.0
Tripod pinch	3.50 (2.60–4.50)	2.70 (1.80–4.30)	0.124	0.0
Jamar	20.30 (13.30–24.80)	20.90 (13.80–25.20)	0.778	0.0
Palmar abduction	46.00 (39.00–56.00)	45.00 (42.00–53.00)	0.766	0.0
Kapandji opposition	10.00 (9.00–10.00)	10.00 (8.00–10.00)	0.946	1.2

a Values are median (interquartile range).

b Hand measurements after matching. Patients who underwent revision surgery (disc removal) are included in this analysis.

c Five missing.

d Six missing.

e Significant.

**Table 4.** Patient-Reported Outcome Measures^a^

PROMs	PDI after Matching	LRTI after Matching	*P*	Missing Measurements, %
No. of patients	62	31		
PRWHE				
Total	16.50 (2.12–54.75)	19.50 (5.12–41.38)	0.641	7.5
Pain	11.00 (0.00–27.25)	5.50 (2.25–25.00)	0.727	5.4
Function	7.00 (1.50–23.88)	13.50 (1.38–23.50)	0.509	7.5
MHQ				
Total	74.30 (62.05–90.15)	73.10 (56.50–90.00)	0.429	5.4
Satisfaction	79.20 (54.20–100.00)	75.00 (45.80–95.80)	0.601	6.5
Aesthestics	100.00 (78.15–100.00)	87.50 (68.75–100.00)	0.009^b^	5.4
Pain	75.00 (55.00–95.00)	62.50 (40.00–95.00)	0.364	5.4
Work	70.00 (47.50–100.00)	80.00 (40.00–95.00)	0.964	5.4
ADL average	80.70 (60.90–94.45)	81.80 (65.40–93.90)	0.756	5.4
ADL treated hand	70.00 (50.00–90.00)	70.00 (50.00–75.00)	0.152	5.4
Satisfaction with result	9.50 (7.00–10.00)	9.00 (6.00–10.00)	0.649	5.4

ADL, activities of daily living; MHQ, Michigan Hand Outcomes Questionnaire; PRWHE, Patient-Rated Wrist/Hand Evaluation.

a Values are median (interquartile range).

b Significant.

**Table 5.** Thumb Length on Radiology^a^

Radiology Thumb Length	PDI after Matching	LRTI after Matching	*P*	Missing Measurements, %
Patients, *n*	62	26		
Thumb length, preoperative	58.2 (55.4–61.6)	57.6 (54.8–60.4)	0.425	16.6
Ratio, preoperative	1.97 (1.90–2.01)	1.94 (1.87–2.01)	0.767	16.6
Thumb length, immediate follow-up	56.60 (54.10–60.20)	50.60 (49.20–52.60)	<0.001	19.4
Ratio, immediate follow-up	1.90 (1.83–2.01)	1.79 (1.70–1.84)	0.001	18.3
Thumb length, LTFU	53.90 (51.38–57.70)	47.95 (45.27–50.82)	<0.001	26.9
Ratio, LTFU	1.83 (1.76–1.91)	1.65 (1.58–1.77)	<0.001	26.9

LTFU, long-term follow-up.

a Values are median (interquartile range).

**Table 6.** Complications

Complications	No. (%)	Revision
PDI		
Disc in situ	54 (87.1)	No revision
STT arthritis	5 (8.1)	Disc removal, converted to trapeziectomy with LRTI
Persisting pain, no cause	3 (4.8)	Disc removal, converted to trapeziectomy with LRTI
LRTI		
LRTI in situ	26 (83.9)	No revision
Infection	2 (6.5)	Incision and drainage, antibiotics, nonoperative therapy
CRPS	3 (9.7)	Hand therapy and medication

CRPS, complex regional pain syndrome; STT, scaphotrapeziotrapezoidal.

## DISCUSSION

This long-term follow-up study comparing PDI CMC thumb arthroplasty with trapeziectomy combined with LRTI showed stronger key and tip pinch strength at a minimum 5-year follow-up with PDI. Both thumb length and thumb height ratio were better preserved in the PDI group. In addition, the PDI-treated patients were more satisfied with the aesthetic result and scored better on activities of daily living for the treated hand in comparison with the LRTI group. Comparable complication rates were observed.

In an earlier study, Oh et al.^22^ found, after 2 years of follow-up, a stronger key pinch for patients treated with PDI compared with those treated with trapeziectomy combined with LRTI. In our study, we found that even after a follow-up of 8 years, key pinch was still better preserved for the PDI group compared with that of the LRTI group, suggesting that that the better preservation of key pinch strength observed after PDI is sustainable.

In our study, we used key pinch as primary outcome, as key pinch is affected in early CMC-1 osteoarthritis.^31^ Compared with the LRTI group, patients of the PDI group had greater key and tip pinch postoperatively. In contrast, earlier long-term follow-up studies comparing different surgical techniques for CMC-1 osteoarthritis did not show any difference in pinch strength postoperatively. Gangopadhyay et al.^32^ compared 3 different techniques: simple trapeziectomy, trapeziectomy with tendon interposition, and trapeziectomy with tendon interposition and ligament reconstruction in 5- to 18-year follow-up. The authors did not find any difference between the techniques in tip or key pinch and concluded that additional techniques are not beneficial to simple trapeziectomy. Brennan et al.^33^ concluded that an additional LRTI is of no added value to trapeziectomy on tip or key pinch at long-term follow-up, and therefore concluded that more expensive treatment options for CMC-1 osteoarthritis are not indicated, as satisfaction is already high after trapeziectomy. Furthermore, it was concluded that, based on the literature, thumb height maintenance has no influence on strength. However, previous studies analyzing thumb height in relation to strength only analyzed techniques with loss of thumb height without evaluating techniques with proven thumb height maintenance,^34,35^ such as the PDI.^14,36^ In our study, we found both stronger key and tip pinch in the PDI compared with the LRTI group, and more thumb height maintenance after PDI, favoring PDI over LRTI in case of CMC thumb arthritis without involvement of the STT joint.

The high costs of the pyrocarbon disc are a disadvantage of the PDI technique. The costs of the disc may be counterbalanced with improved results on strength and faster return to work. However, we did not evaluate return to work in this study. Therefore, we could not perform a proper cost–benefit analysis comparing the techniques.

Our results are in correspondence with those of other studies concerning other thumb implants showing good outcomes and high satisfaction rates. Data from 5-year follow-up of the Arpe prosthesis in 121 thumbs of 116 patients showed excellent scores on the Quick Disabilities of the Arm, Shoulder, and Hand questionnaire and good key pinch, grip strength, and ROM in comparison with simple trapiezectomy.^37^ Survival analysis showed a high survival rate (95%) with end point indication for revision. Indication for the use of prostheses for CMC thumb joint arthritis is under debate, but our results and those of Cootjans et al.^37^ show that there could be a place for the use of prostheses. The advantage of the pyrocarbon disc over the total implant is the relative low risk for subluxation and no risk of fracturing of the stem or loosening of the trapezium component.

Various surgical treatment strategies are used for CMC thumb joint osteoarthritis, although none has been proven to be superior.^5,38^ Therefore, simple trapeziectomy is considered the standard, because this technique has fewer complications compared with other common techniques.^5,39^ The complication ratio after PDI was comparable with LRTI based on this study (12.9%; Table 5). Eight PDI-treated patients (12.9%) underwent revision surgery with disc removal because of pain caused by progressive STT arthritis or without a known cause. In 3 patients, the disc was removed before the first year of follow-up (in 2 of these patients because of STT arthritis, which should have been addressed at the initial operation). In 3 patients, STT arthritis caused pain after 2 years of follow-up (2, 2.7, and 7.7 years, respectively). In case of persisting pain, it was decided to remove the disc and perform a full trapeziectomy with LRTI (after 10, 12, and 13 months). In 4 patients, the outcomes after disc removal, full trapeziectomy, and LRTI remained poor on hand measurements and PROMs. In the LRTI group, 2 patients had a postoperative infection and underwent operative incision and drainage. On long-term follow-up, no revision surgery was performed in the LRTI group. The survival rate of the PDI technique reported in the literature is 91%.^14^ The survival rate in this study was lower, which was attributable to matching in this cohort. After matching, a relatively high number of patients after disc removal was selected. Nowadays, STT osteoarthritis is more strictly diagnosed preoperatively, reducing the revision rate of PDI.

The following limitations need to be considered when interpreting the results. First, the sample size of the LRTI group is limited, mostly because of patients’ and surgeons’ preferences for PDI treatment in Eaton-Glickel stage II or III. Our post hoc analyses showed that we were primarily able to detect moderate effect sizes; therefore, we may have missed small differences between the 2 groups. However, these differences may not be clinically relevant. Second, our results were analyzed based on PSM. In this type of analysis, one is only able to match upon the smallest group within the study and can only match on variables without missing values, which are inevitable in retrospective research. Study patients may end up not being matched, and excluded for further analysis, which may affect the generalizability of our results. Nevertheless, PSM is a commonly accepted analysis to mimic a randomized controlled trial in observational study cohorts and, as a result, of value in treatment studies.^40^ The sound analysis technique and long follow-up data of this study are therefore of value in the scientific field of surgical treatment of CMC-1 joint osteoarthritis. Third, preoperative hand measurements were not available in our study because of their observational nature. Future prospective studies should include preoperative hand measurement data to determine the exact gain or loss of hand function postoperatively. In addition, we did not assess return to work, limiting cost-effectiveness analysis of implant surgery compared with other techniques, such as simple trapeziectomy.

## CONCLUSIONS

The current study showed that even after long-term follow-up, key pinch, tip pinch, and thumb length are better preserved after pyrocarbon interposition arthroplasty compared with simple trapeziectomy combined with an LRTI for CMC-1 joint osteoarthritis. The techniques have comparable patient-reported outcomes, ROM outcomes, and complications.

## DISCLOSURE

The authors have no financial interest in any of the products, devices, or drugs mentioned in this article, and have no conflicts of interest to report.
